# Relational energy and coping flexibility among Chinese university students: the mediating role of optimism and the moderating influence of cognitive reappraisal

**DOI:** 10.3389/fpsyg.2026.1760606

**Published:** 2026-04-10

**Authors:** Dongliang Liu, Juan Liu

**Affiliations:** 1 School of Marxism, Shanxi Vocational University of Engineering Science and Technology, Jinzhong, China; 2 School of Educational Science, Northwest Normal University, Lanzhou, China

**Keywords:** cognitive reappraisal, coping flexibility, moderated mediation, optimism, PLS-SEM, positive psychology, relational energy

## Abstract

**Introduction:**

This study examines how relational energy influences coping flexibility among university students, integrating optimism as a mediating mechanism and cognitive reappraisal as a key boundary condition. Drawing on social resource and positive psychology perspectives, the model proposes that energized interpersonal interactions foster optimism, which in turn enhances students’ ability to flexibly adapt their coping strategies.

**Methods:**

A face-to-face survey was conducted with 500 university students in China, yielding 436 valid responses. Partial least squares structural equation modeling (PLS-SEM) was employed to assess both the measurement and structural models.

**Results:**

The findings indicate that relational energy is positively associated with both optimism and coping flexibility. Optimism partially mediates the relationship between relational energy and coping flexibility. Furthermore, cognitive reappraisal significantly strengthens the relationship between relational energy and optimism and amplifies the indirect effect of relational energy on coping flexibility through optimism.

**Discussion:**

These results highlight the complementary roles of interpersonal vitality and adaptive emotion regulation in shaping students’ psychological adaptability. The study contributes to the relational energy literature by extending its relevance to academic contexts and underscores the importance of fostering both social and cognitive resources to promote student resilience.

## 1 Introduction

The increasing complexity of academic life exposes university students to escalating psychological demands, making it essential to understand the resources that help them navigate stress with adaptability. Contemporary research acknowledges that students must regulate not only academic pressures but also the interpersonal and emotional challenges inherent in university environments (Arslan, 2024; Podiya et al., 2025). Within this context, scholars emphasize the importance of internal and external resources that enhance students’ capacity to cope with stressors in flexible and constructive ways (Chaudhry et al., 2024). Although numerous studies outline cognitive and emotional predictors of adaptive coping (Brites et al., 2024; Najwakhida and Aeni, 2025), the question of how social and relational experiences shape students’ psychological functioning remains insufficiently addressed. In particular, while coping flexibility research has largely focused on individual cognitive or emotional resources (Kaynak et al., 2024; Wang et al., 2025), the potential role of interpersonal energy generated through social interactions remains relatively underexplored. This gap highlights the need for a more integrated understanding of coping flexibility that accounts for both interpersonal and intrapersonal influences.

Building on this broader concern, recent scholarship increasingly recognizes that the social environment provides more than functional support; it also fuels psychological energy that strengthens individuals’ capacity for engagement, resilience, and goal-directed behavior (Chaudhry et al., 2024; Ding et al., 2024). A growing stream of work suggests that energizing social interactions generate emotional uplift, broaden attentional resources, and stimulate positive psychological states (Fang et al., 2025; Kim et al., 2024; Podiya et al., 2025). This perspective aligns with the concept of relational energy, defined as the heightened psychological vitality individuals experience during interpersonal interactions that leave them feeling motivated, mentally alert, and energized (Owens et al., 2016). Importantly, relational energy differs from related constructs such as social support or interpersonal climate because it reflects the energizing psychological effect of interactions rather than the provision of assistance or the general tone of social environments (Cabrera et al., 2025). Such insights signal that students’ day-to-day interactions may serve as an important, yet overlooked, source of psychological resourcefulness. However, despite evidence linking interpersonal dynamics to enhanced emotional functioning, limited research explores whether the vitality derived from social exchanges contributes to students’ expectations about the future or their ability to adapt their coping strategies. This gap invites a closer look at how relationally uplifting experiences may lay the groundwork for adaptive cognitive and behavioral responses.

Extending this line of inquiry, research in positive psychology consistently shows that individuals’ future-oriented beliefs exert powerful influence over how they perceive challenges and respond to demands. Optimistic expectations are associated with greater perseverance, reduced emotional distress, and more flexible approaches to coping (Amiri Dahaj et al., 2024). In addition, emerging evidence suggests that positive cognitive states may be strengthened through affirming and energizing interpersonal experiences (Galindo and Sanders, 2022; Rezaei and Khosroshahi, 2018), raising important theoretical possibilities. From the perspective of broaden-and-build theory (Fredrickson, 2004), positive affective experiences generated through energizing interpersonal interactions may broaden individuals’ cognitive and attentional resources, encouraging more expansive thinking about future possibilities and challenges. When individuals experience relational energy, the accompanying psychological vitality may therefore stimulate more positive expectations about outcomes, thereby fostering optimism. If relationally rich interactions stimulate broader and more constructive thinking patterns, then optimism may serve as a key psychological conduit through which interpersonal vitality translates into adaptive coping processes. Yet, the literature remains largely silent on how these interpersonal and cognitive pathways interconnect within university settings, leaving a meaningful conceptual space open for investigation.

Alongside interpersonal and cognitive influences, emotional self-regulation represents another critical mechanism shaping students’ capacity to respond effectively to stress. Strategies such as cognitive reappraisal allow individuals to reinterpret adverse circumstances and potentially generate more adaptive emotional responses (Rodriguez et al., 2020). Research indicates that students who frequently engage in reappraisal exhibit stronger resilience, more constructive coping behavior, and greater psychological stability (Abraham et al., 2023). When considered alongside interpersonal and cognitive perspectives, reappraisal emerges as a potentially important moderator that may enhance the psychological gains students derive from energizing social interactions. Individuals with strong reappraisal tendencies may therefore be better positioned to internalize interpersonal vitality in ways that foster optimism and adaptive coping. Despite these theoretical possibilities, the literature has yet to fully examine emotion regulation as a boundary condition within relational and cognitive pathways.

Taken together, prior scholarship emphasizes the importance of examining multiple intersecting mechanisms—interpersonal, cognitive, and emotional—that jointly shape adaptive coping in academic contexts. Yet, existing studies rarely integrate these dimensions into a cohesive explanatory framework. To address this gap, the present study proposes a moderated mediation model in which relational energy enhances coping flexibility both directly and indirectly through optimism, while cognitive reappraisal strengthens the relational energy–optimism pathway and amplifies its downstream effects. By integrating interpersonal vitality (relational energy), cognitive expectations (optimism), and emotion regulation processes (cognitive reappraisal) within a single conditional process framework, this study extends positive psychology research by demonstrating how social, cognitive, and regulatory resources may jointly contribute to coping flexibility among university students. By situating these constructs within a unified framework, the study advances theoretical understanding of how students mobilize psychological resources in response to academic stress and offers new insight into how interpersonal vitality, cognitive expectations, and emotion regulation jointly support adaptive functioning.

### 1.1 Hypotheses

#### 1.1.1 Relationship between relational energy and coping flexibility

Relational energy represents the psychological resourcefulness and vitality individuals gain from uplifting interpersonal interactions, allowing them to feel motivated, engaged, and emotionally strengthened in their daily functioning (Braha and Karabulut, 2023). Coping flexibility, in contrast, refers to the ability to assess stressful demands, modify coping strategies, and shift responses when existing approaches prove ineffective, enabling individuals to adaptively manage complex or evolving stressors (Zimmer-Gembeck, 2021). These two constructs intersect meaningfully within contemporary psychological frameworks, as social resource theories propose that energizing interpersonal exchanges broaden individuals’ emotional and cognitive capacities, thereby facilitating more adaptive behavioral responses to stress (Creed et al., 2022). In addition, Jiang et al. (2024) argue that relational energy enhances vitality and mental agility, which are foundational for flexible coping. Furthermore, there is growing evidence that energizing social interactions promote cognitive clarity and broadened attentional resources, both of which support adaptive coping strategies (Aslamina et al., 2024). Besides this, socially induced positive affect is known to increase individuals’ willingness to reevaluate coping options and engage in more effective problem-solving behaviors (Sining et al., 2022). Prior studies also suggest that interpersonal vitality contributes to greater psychological resilience and stress tolerance, creating conditions under which flexible coping is more likely to occur (Pineda et al., 2022). Likewise, energized social environments stimulate motivation and emotional stability, which facilitate the reassessment and adjustment of coping strategies during challenging circumstances (Loureiro et al., 2024). Simultaneously, research on social connectedness indicates that interpersonal engagement enhances adaptive coping by providing emotional reinforcement and cognitive support (Wang, 2025). Taken together, there is a growing consensus that interpersonal sources of psychological energy operate as catalysts for adaptive coping processes, supporting the idea that relationally energized individuals can more readily shift coping strategies when demands change (Huang et al., 2024). Building on these theoretical and empirical insights, the present study proposes the following hypothesis:

*H1*: Relational energy positively influences coping flexibility.

#### 1.1.2 Relationship between relational energy and optimism

The relationship between relational energy and optimism can be meaningfully postulated by recognizing that energizing social interactions have the potential to stimulate positive future-oriented expectations within individuals. Optimism, typically defined as a generalized belief that favorable outcomes are more likely to occur than unfavorable ones, reflects a cognitive orientation that guides how individuals interpret challenges and anticipate their capacity to handle them (Amiri Dahaj et al., 2024). Positive psychology frameworks suggest that interpersonal experiences rich in vitality and emotional uplift may foster such optimistic outlooks by enhancing individuals’ sense of capability and reinforcing perceptions of support (Rezaei and Khosroshahi, 2018). In addition, Cabrera et al. (2025) posit that relational energy heightens psychological resourcefulness, which naturally broadens individuals’ expectations about what is possible in the future. Furthermore, energized interactions enhance affective states and cognitive flexibility, both of which are known predictors of optimism (Liu et al., 2023). Besides this, social environments that convey affirmation and enthusiasm often cultivate elevated perceptions of hope and positive expectancy, as demonstrated by evidence linking interpersonal vitality with strengthened motivational states (Yung et al., 2021). Research in academic and organizational settings also shows that supportive and energizing relationships promote positive cognitive appraisal patterns, thereby increasing optimistic thinking (Monehin and Diers-Lawson, 2024). Likewise, studies in wellbeing research reveal that individuals who experience higher levels of relational engagement tend to adopt more favorable interpretations of setbacks and future possibilities (Crane et al., 2022). A growing consensus stipulates that emotionally energizing interactions stimulate upward cognitive spirals that reinforce optimism, functioning as a psychological amplifier that strengthens one’s belief in positive future outcomes (Merati, 2021). Drawing on these converging insights, it is reasonable to infer that relational energy serves as an interpersonal catalyst that fosters heightened optimism among students.

*H2*: Relational energy positively influences optimism.

#### 1.1.3 Mediating role of optimism

The mediating role of optimism can be grounded in theoretical perspectives suggesting that positive expectations about the future serve as a key psychological mechanism through which interpersonal experiences influence adaptive functioning. Optimism, reflecting a generalized belief that favorable outcomes are attainable, shapes how individuals evaluate challenges, select coping strategies, and persevere under stress (Monehin and Diers-Lawson, 2024). When viewed through this lens, relational energy may operate as an interpersonal precursor that enhances optimism, which then facilitates coping flexibility. In addition, Fredrickson’s broaden-and-build theory posits that positive cognitive states expand individuals’ thought–action repertoires, enabling them to adaptively modify coping strategies when demands shift (Fredrickson, 2004). Furthermore, relationally uplifting interactions heighten positive affect, strengthen cognitive clarity, and reinforce perceptions of control, all of which are known antecedents of optimistic thinking (Goodwin and Williams, 2023). Besides this, energized social exchanges promote psychological vitality and broaden cognitive framing, increasing individuals’ readiness to interpret stressors in constructive ways (Liu et al., 2021). Prior research similarly demonstrates that optimism acts as a pathway through which social resources influence resilience, emotional stability, and coping outcomes (Tras et al., 2021). Simultaneously, studies show that individuals who experience higher interpersonal engagement tend to develop stronger future-oriented beliefs that support more flexible coping responses (Armeli et al., 2025). Together, this body of evidence suggests that optimism may explain how the motivational and emotional benefits of relational energy translate into adaptive coping processes. As such, optimism is theoretically positioned as a central mechanism that converts interpersonal vitality into flexible coping behavior.

*H3*: Optimism mediates the relationship between relational energy and coping flexibility.

#### 1.1.4 Moderating role of cognitive reappraisal

The moderating role of cognitive reappraisal can be proposed by recognizing that individuals’ emotion regulation tendencies shape how they internalize and interpret interpersonal experiences. Cognitive reappraisal refers to the deliberate process of reframing the meaning of a situation to alter its emotional impact, enabling individuals to generate more adaptive and constructive responses during stress (Stover et al., 2024). According to Riepenhausen et al. (2022), this strategy is widely regarded as an adaptive regulatory mechanism that enhances resilience, stabilizes affect, and promotes positive cognitive evaluations. Within this framework, cognitive reappraisal is expected to strengthen the influence of relational energy on optimism because individuals who habitually reinterpret situations are more capable of transforming energizing social interactions into sustained positive expectations. In addition, research indicates that reappraisal fosters psychological openness and cognitive flexibility, allowing individuals to more effectively absorb and reframe uplifting interpersonal cues (Marciniak et al., 2024). Furthermore, those with strong reappraisal tendencies exhibit greater emotional stability, which amplifies the benefits of supportive or energizing relationships (Nakhostin-Khayyat et al., 2024). Besides this, reappraisal is associated with higher levels of positive affect and future-oriented thinking, both of which reinforce the development of optimistic belief patterns (Hayatbini et al., 2021). Likewise, studies show that individuals who regulate their emotions adaptively are more responsive to relational signals that convey vitality and support (Liu et al., 2023). Simultaneously, evidence suggests that emotion regulation moderates the extent to which social resources enhance psychological outcomes, implying that interpersonal energy may be especially impactful when paired with adaptive regulatory strategies (Walker et al., 2022). Taken together, this body of research suggests that cognitive reappraisal enhances the cognitive impact of relational energy, making optimistic outlooks more likely to emerge among individuals with strong reappraisal capacity.

*H4*: Cognitive reappraisal moderates the relationship between relational energy and optimism such that the relationship is stronger at higher levels of cognitive reappraisal.

#### 1.1.5 Moderated mediation model

The combined logic of the mediation and moderation hypotheses suggests a broader conditional process through which relational energy influences coping flexibility. While optimism is theoretically positioned as a mediating mechanism that converts energized interpersonal experiences into adaptive coping responses, the strength of this mediating pathway is expected to vary depending on individuals’ emotion regulation tendencies. Cognitive reappraisal, as an adaptive form of emotional reframing, enhances individuals’ ability to reinterpret interpersonal cues in constructive ways, thereby amplifying the positive psychological effects of relational energy (Hayatbini et al., 2021). In addition, Fredrickson’s broaden-and-build framework proposes that individuals who generate positive reinterpretations are more likely to translate social uplift into broadened future-oriented thinking (Fredrickson, 2004). When these perspectives are integrated, it becomes evident that individuals with strong reappraisal tendencies are better positioned to transform relational energy into heightened optimism, which subsequently facilitates more flexible coping. Furthermore, prior studies indicate that emotion regulation enhances the cognitive benefits associated with supportive social experiences (Liu et al., 2023; Rodriguez et al., 2020), while others show that optimism-based pathways are particularly sensitive to regulatory processes that govern emotional meaning-making (Stover et al., 2024). Besides this, research on conditional indirect effects demonstrates that psychological mechanisms often operate differently across individuals based on their regulatory capabilities, suggesting that cognitive reappraisal may magnify the interpersonal-to-cognitive-to-behavioral chain identified in this study (Clark, 2022). Taken together, these insights support a moderated mediation logic in which cognitive reappraisal strengthens the mediating effect of optimism, making the indirect influence of relational energy on coping flexibility more pronounced for students who habitually engage in reappraisal.

*H5*: Cognitive reappraisal moderates the indirect effect of relational energy on coping flexibility through optimism such that the mediated relationship is stronger at higher levels of cognitive reappraisal.

**FIGURE 1:**
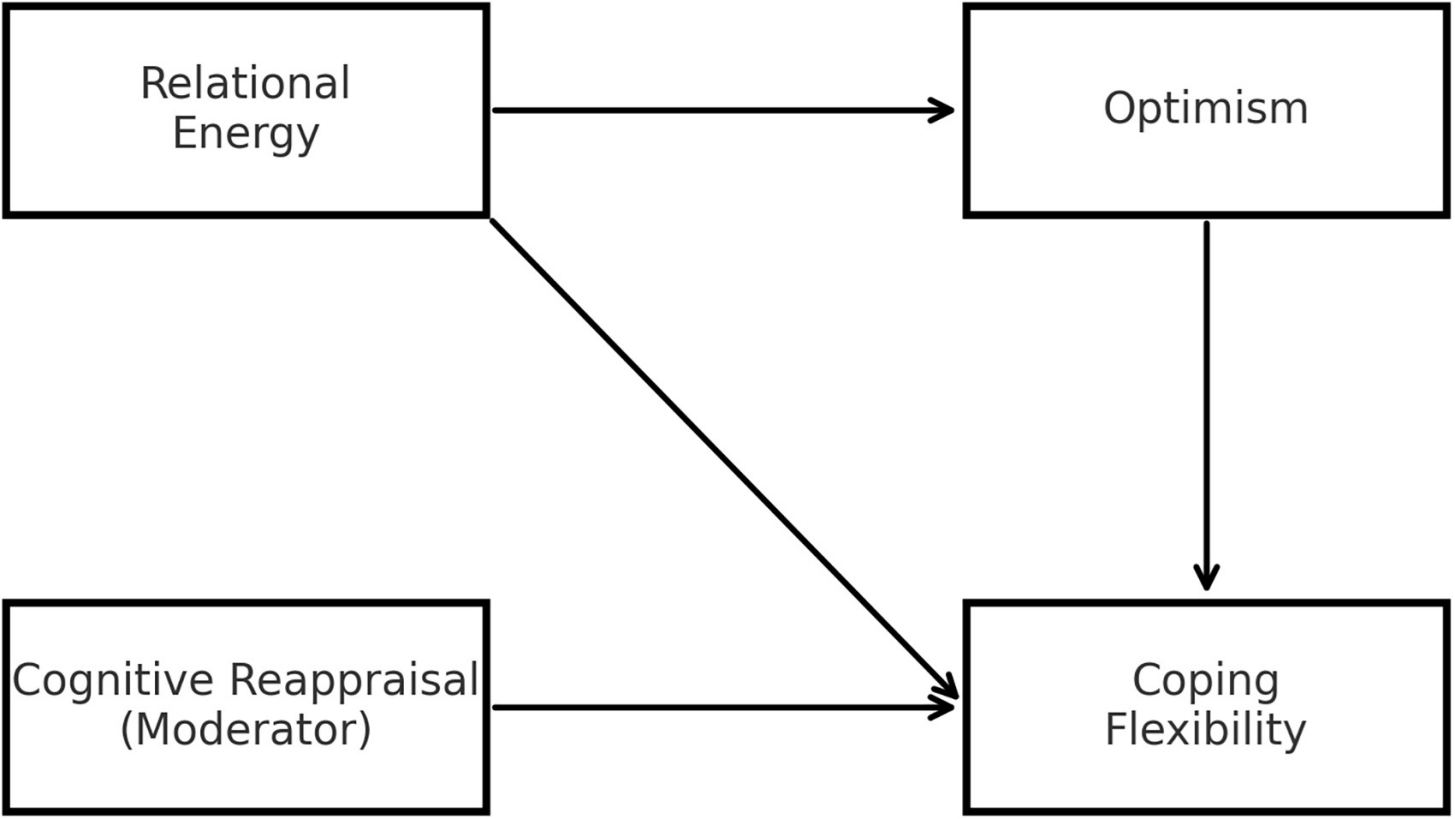
Conceptual model.

Figure 1 presents the proposed conceptual model.

## 2 Methodology

The study uses a quantitative, questionnaire-based survey design to examine the proposed research model among university students in China. A total of 500 questionnaires were distributed using a face-to-face, in-person administration approach, which ensures higher response accuracy and allows respondents to seek clarification when needed. This method is particularly suitable for studies requiring careful interpretation of psychological constructs, increasing the likelihood of complete and reliable data.

A non-probability purposive sampling technique is employed, as the study specifically targets students enrolled in full-time degree programs who have regular academic and interpersonal engagement on campus. This sampling approach is justified because the constructs under investigation—relational energy, optimism, cognitive reappraisal, and coping flexibility—require respondents who actively participate in social and academic interactions, making university students an appropriate and relevant population. Purposive sampling is therefore considered suitable for identifying participants who are likely to experience the interpersonal dynamics central to the study constructs. However, because this approach does not provide equal selection probability across the broader population of Chinese university students, the findings should be interpreted with appropriate caution regarding generalizability.

Participants were approached on campus in common study areas, cafeterias, and classroom buildings. Each respondent received a printed survey booklet containing a brief cover letter explaining the purpose of the study, emphasizing the voluntary nature of participation, assuring confidentiality, and clarifying that the data would be used solely for academic research. Out of the 500 questionnaires distributed, 468 were returned, and after screening for incomplete, patterned, or invalid responses, 436 fully usable questionnaires were retained and included in the final analysis. The excluded questionnaires primarily contained missing responses or patterned answering that indicated insufficient response quality. Because the proportion of excluded surveys was relatively small and resulted mainly from data quality screening rather than systematic nonresponse, the likelihood of significant attrition bias is considered limited.

The demographic profile of the respondents reflects diverse student characteristics. Approximately 56 percent of the sample is female and 44 percent is male, reflecting a typical gender distribution in many Chinese university programs. In terms of age, 18 percent of the students fall between 18 and 19 years, 47 percent between 20 and 21 years, 28% between 22 and 23 years, and the remaining 7 percent are 24 years or older. Academic standing shows that 41% are first- or second-year students, 39 percent are in their third or fourth year, and 20 percent are enrolled in postgraduate programs. Regarding study discipline, 32 percent come from business and management fields, 27 percent from social sciences, 22 percent from engineering or technology programs, and 19 percent from arts and humanities. Additionally, 64 percent reside in on-campus dormitories, while 36 percent live off campus.

### 2.1 Measures

The measures used in this study are inspired by well-established instruments adopted in previous empirical research. All items are rated on a five-point Likert scale ranging from 1 = *strongly disagree* to 5 = *strongly agree*, allowing participants to indicate the extent to which each statement reflected their typical experiences. Because the study was conducted among Chinese university students, all survey items were translated into Chinese using a back-translation procedure to ensure conceptual and linguistic equivalence (Brislin, 1970). Two bilingual researchers independently translated the items into Chinese and subsequently back-translated them into English. Any discrepancies were discussed and resolved to maintain semantic consistency with the original measurement instruments.

#### 2.1.1 Relational energy

Relational energy is measured using the five-item Relational Energy Scale developed by Owens et al. (2016), which assesses the degree to which interpersonal interactions generate feelings of vitality and psychological resourcefulness. A representative item includes *“I feel invigorated when I interact with the people around me.”*

#### 2.1.2 Optimism

Optimism is captured using the Life Orientation Test–Revised (LOT-R; Scheier et al., 1994), a six-item scale widely used to assess generalized positive expectations about the future. Sample items include *“In uncertain times, I usually expect the best”* and the reverse-coded statement *“If something can go wrong for me, it will.”*

#### 2.1.3 Cognitive reappraisal

Cognitive reappraisal tendency, serving as the moderating variable, is assessed through the Cognitive Reappraisal subscale of the Emotion Regulation Questionnaire (Gross and John, 2003). This six-item measure evaluates individuals’ habitual reliance on reframing emotional situations, exemplified by items such as *“I control my emotions by changing the way I think about the situation I’m in.”*

#### 2.1.4 Coping flexibility

Finally, coping flexibility is measured using the 10-item Coping Flexibility Scale (Kato, 2012), which reflects individuals’ ability to evaluate coping strategies and adjust them when needed. Example items include *“I am aware of how successful or unsuccessful my attempts to cope with stress have been”* and *“When a stressful situation has not improved, I try to think of other ways to cope with it.”*

### 2.2 Analysis

Partial least squares structural equation modeling (PLS-SEM) is used in this study because it is well suited for predictive, theory-building research and accommodates complex models involving multiple mediating and moderating relationships (Hair et al., 2021). PLS-SEM is particularly appropriate for the present study because the research model includes both mediation and interaction effects, which increase model complexity and benefit from a variance-based estimation approach focused on maximizing explained variance in endogenous constructs (Hair et al., 2021). Further, PLS-SEM should be used when the goal is to maximize explained variance in key outcomes and when constructs are measured using multi-item reflective indicators. Following established guidelines, the analysis first assesses the measurement model, including indicator reliability, internal consistency reliability (Cronbach’s alpha and composite reliability), convergent validity through average variance extracted, and discriminant validity using both the Fornell–Larcker criterion and the heterotrait–monotrait ratio. Next, the structural model is evaluated by estimating path coefficients, coefficient of determination (*R*^2^), predictive relevance (*Q*^2^), and effect sizes (*f*^2^). Mediation and moderation hypotheses are examined using bias-corrected bootstrapping with 5,000 resamples, providing robust inference for indirect and interaction effects (Preacher and Hayes, 2008). Variance inflation factor (VIF) values are also assessed to ensure the absence of multicollinearity among predictor constructs. These procedures align with best practices for conducting rigorous PLS-SEM analysis in behavioral and social science research.

In addition, the conceptual structure of the proposed framework corresponds to a first-stage moderated mediation model that is equivalent to PROCESS Model 7 as described by Hayes (2018). In this configuration, relational energy serves as the independent variable, optimism functions as the mediating mechanism linking relational energy to coping flexibility, and cognitive reappraisal moderates the first-stage path from relational energy to optimism. Consequently, the indirect effect of relational energy on coping flexibility through optimism is conditional upon the level of cognitive reappraisal.

Although PROCESS models are typically estimated using regression-based procedures, the present study operationalizes this conditional process framework within the PLS-SEM environment. Specifically, the moderation effect is modeled through the construction of an interaction term between relational energy and cognitive reappraisal using the product indicator approach recommended for variance-based SEM (Hair et al., 2021). This interaction construct is incorporated into the structural model to predict optimism. Conditional indirect effects are subsequently examined through bias-corrected bootstrapping with 5,000 resamples, which allows the estimation of the indirect relationship between relational energy and coping flexibility through optimism at different levels of cognitive reappraisal. This procedure preserves the conceptual logic of Hayes’ conditional process modeling while taking advantage of the predictive capabilities of PLS-SEM for estimating complex models that include mediation and interaction effects simultaneously.

### 2.3 Control

To address potential confounding influences, additional analyses including gender, age, and academic level as control variables were conducted. The inclusion of these covariates did not materially alter the main structural relationships, indicating that the hypothesized effects remain robust.

### 2.4 Common method bias assessment

The study collected all variables were through self-report questionnaires in a single survey session, therefore common method variance (CMV) was assessed using statistical diagnostics. Harman’s single-factor test indicated that the first factor accounted for 34.2% of the total variance, which is below the recommended threshold of 50%, suggesting that CMV is unlikely to substantially influence the results (Podsakoff et al., 2003). In addition, full collinearity variance inflation factors were examined following Kock (2015). The analysis results are reported in the subsequent section.

## 3 Results

Table 1 illustrates the collinearity statistics for all predictor constructs using variance inflation factor (VIF) values. All VIF values fall well below the recommended upper threshold of 3.3, indicating no issues of multicollinearity among the constructs and confirming that shared variance does not distort the structural paths in the model (Hair et al., 2021). This supports the adequacy of the data for subsequent structural analyses.

**TABLE 1:** Collinearity statistics (VIF).

Construct	CF	CR	OPT	RE
CF	1.031		1.028	
CR				
OPT	1.285		1.022	
RE	1.253			

Table 2 demonstrates the reliability and convergent validity of all constructs. Factor loadings exceed the acceptable cutoff of 0.70 for the majority of items, while Cronbach’s alpha and composite reliability values for all constructs remain above 0.80, satisfying internal consistency criteria (Hair et al., 2021). Additionally, the average variance extracted (AVE) values for each construct are above 0.50, supporting adequate convergent validity. The few items removed during refinement, such as CF10 and CR4, further improve the psychometric quality of the scales, reinforcing the robustness of the measurement model.

**TABLE 2:** Constructs’ reliability and validity.

Items	Loadings	CA	rho_a	rho_c	AVE
Cognitive flexibility		0.919	0.921	0.933	0.608
CF1	0.746	0.832	0.894	0.877	0.591
CF2	0.741				
CF3	0.778				
CF4	0.803				
CF5	0.748				
CF6	0.795				
CF7	0.794				
CF8	0.777				
CF9	0.828				
CF10	Dropped				
Cognitive reappraisal					
CR1	0.877	0.904	0.907	0.926	0.676
CR2	0.843				
CR3	0.789				
CR4	Dropped				
CR5	0.670				
CR6	0.634				
Optimism					
OPT1	0.828	0.874	0.875	0.908	0.665
OPT2	0.804				
OPT3	0.805				
OPT4	0.838				
OPT5	0.820				
OPT6	0.839				
Relational energy					
RE2	0.826				
RE3	0.825				
RE4	0.812				
RE5	0.794				
RE1	0.819				

Table 3 presents the discriminant validity results using both the Fornell–Larcker criterion and the heterotrait–monotrait (HTMT) ratios. The square roots of the AVE values, displayed on the diagonal, are higher than corresponding inter-construct correlations, confirming adequate discriminant validity (Fornell and Larcker, 1981). In addition, the HTMT ratios remain well below the conservative threshold of 0.85 (Henseler et al., 2015), further indicating that each construct is empirically distinct and measures a unique conceptual domain.

**TABLE 3:** Discriminant validity.

Construct	Fornell–Larcker				Heterotrait–monotrait			
	CF	CR	OPT	RE	CF	CR	OPT	RE
CF	0.780	0.768	0.822	0.815				
CR	−0.052				0.063			
OPT	0.539	−0.105			0.585	0.113		
RE	0.477	−0.077	0.447		0.530	0.084	0.498	

Table 4 reports the model fit indices. The standardized root mean square residual (SRMR) value is approximately 0.05 for both the saturated and estimated models, which meets the recommended criterion of SRMR < 0.08, demonstrating a good model fit (Henseler et al., 2015). Additional metrics such as d_ULS, d_G, chi-square, and NFI further corroborate acceptable global fit levels, indicating that the model adequately reproduces the empirical correlation matrix.

**TABLE 4:** Model fit.

Fit index	Saturated model	Estimated model
SRMR	0.052	0.051
d_ULS	0.874	0.844
d_G	0.379	0.377
Chi-square	955.966	943.579
NFI	0.851	0.853

To further assess the distinctiveness of the study constructs and the adequacy of the measurement model, a series of confirmatory factor analyses were conducted using covariance-based structural equation modeling in AMOS. Alternative measurement models were compared with the hypothesized four-factor model. As shown in Appendix Table 1, the hypothesized four-factor model demonstrated substantially better fit than the alternative models in which constructs were combined. These results support the discriminant validity of the constructs and provide additional evidence for the adequacy of the proposed measurement structure.

Table 5 provides the results of hypothesis testing. The direct effect of relational energy on coping flexibility (H1) is positive and significant [β = 0.290, *t* = 5.230, *p* < 0.001, 95% CI (0.179, 0.399)], indicating that students who experience higher relational energy demonstrate greater coping flexibility. The pathway from relational energy to optimism (H2) is also strongly supported [β = 0.425, *t* = 6.762, *p* < 0.001, CI (0.299, 0.537)], suggesting that energized interpersonal interactions enhance individuals’ generalized positive expectancies.

**TABLE 5:** Relationship among variables.

Relationship	Path	Confidence intervals		STDEV	*T*	*P*
		2.5%	97.5%			
RE -> CF (*H1*)	0.290	0.179	0.399	0.055	5.230	0.000
RE -> OPT (*H2*)	0.425	0.299	0.537	0.063	6.762	0.000
RE -> OPT -> CF (*H3*)	0.165	0.091	0.244	0.039	4.264	0.000
CR × RE -> OPT (*H4*)	0.116	0.021	0.207	0.058	2.003	0.046
CR × RE -> OPT -> CF (*H5*)	0.102	0.019	0.184	0.050	2.032	0.043

The indirect effect of relational energy on coping flexibility through optimism (H3) is significant [β = 0.165, *t* = 4.264, *p* < 0.001, CI (0.091, 0.244)], confirming optimism as a meaningful psychological mechanism that transmits the benefits of relational energy.

The interaction effect between cognitive reappraisal and relational energy on optimism (H4) is significant [β = 0.116, *t* = 2.003, *p* = 0.046, CI (0.021, 0.207)], indicating that individuals with higher reappraisal tendencies derive stronger optimism from relational energy. Finally, the moderated mediation effect (H5) is supported [β = 0.102, *t* = 2.032, *p* = 0.043, CI (0.019, 0.184)], demonstrating that the indirect effect of relational energy on coping flexibility via optimism becomes stronger at higher levels of cognitive reappraisal. Collectively, these findings provide empirical support for the proposed theoretical framework. Although the moderation and moderated mediation effects are modest in magnitude, such effects are common in psychological models involving interpersonal and cognitive processes (Prentice and Miller, 2016). Even relatively small interaction effects may still have meaningful implications in real-world contexts where multiple psychological resources operate simultaneously to influence students’ coping behavior.

In terms of effect magnitude, the direct relationship between relational energy and optimism represents a moderate effect, while the indirect and conditional effects are comparatively smaller. This pattern is consistent with prior research indicating that psychological and interpersonal processes often exert incremental rather than large effects on behavioral outcomes (Pradhan and Kumar, 2021). In educational contexts (e.g., Reed, 2016), even modest increases in optimism and coping flexibility may be meaningful because they contribute cumulatively to students’ ability to manage academic stress and adapt to changing demands.

**FIGURE 2:**
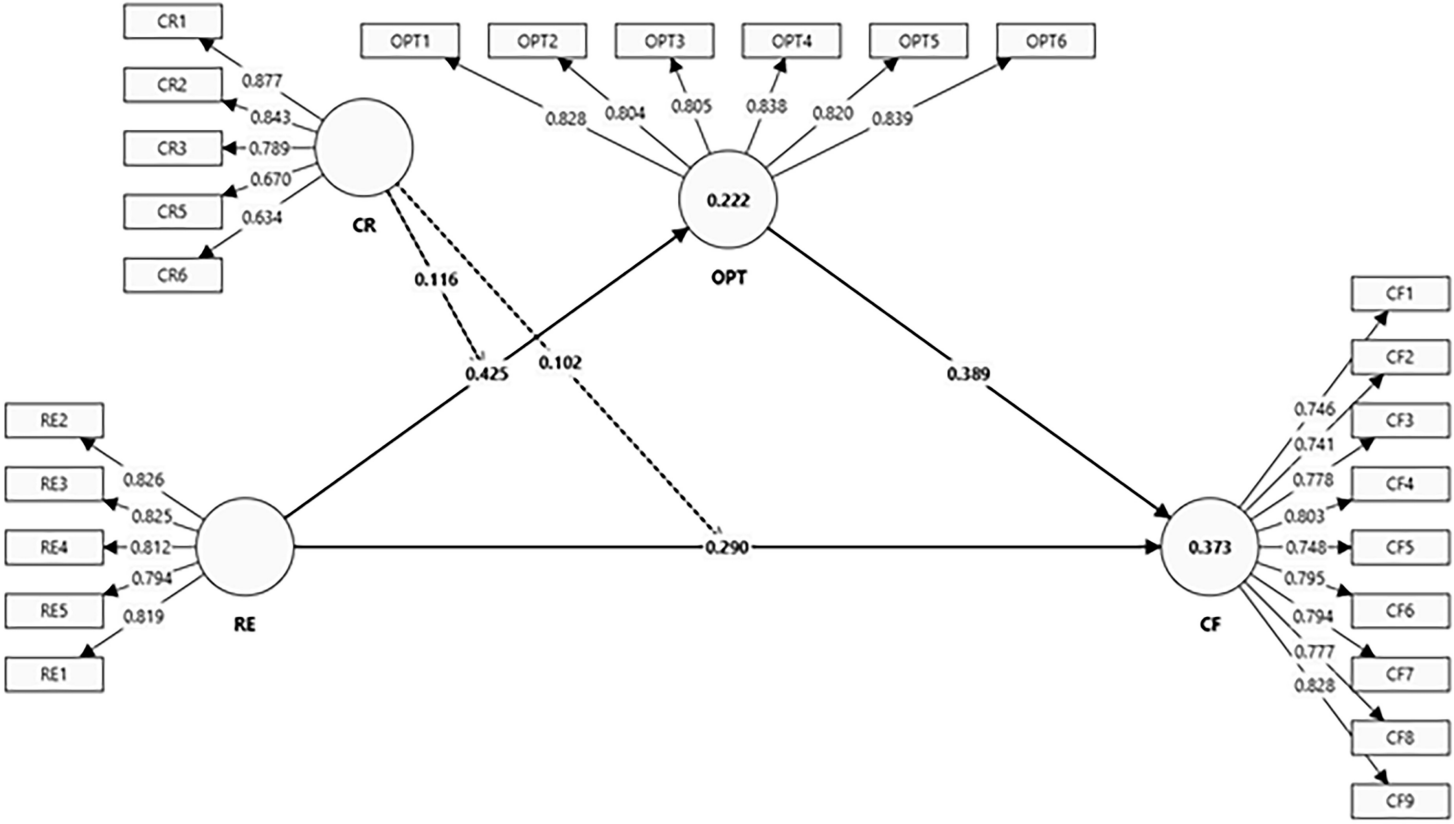
Structural equation model.

Figure 2 visually represents the structural model corresponding to the results reported in Table 5. The diagram illustrates the positive direct paths, the indirect mediation pathway through optimism, and the moderating role of cognitive reappraisal on the relational energy–optimism relationship. This figure helps reinforce the interpretation of the statistical paths by showing how the interplay of relational energy, optimism, and cognitive reappraisal jointly contributes to coping flexibility.

Table 6 presents the predictive relevance results using *Q*^2^ predict along with RMSE and MAE values. The positive *Q*^2^ values for both coping flexibility and optimism exceed zero, which confirms that the model demonstrates meaningful predictive relevance for the endogenous constructs (Hair et al., 2021). RMSE and MAE values also remain within acceptable ranges, indicating good predictive accuracy.

**TABLE 6:** PLS predict LV summary.

Construct	*Q*^2^ predict	RMSE	MAE
CF	0.233	0.888	0.584
OPT	0.200	0.904	0.620

**FIGURE 3:**
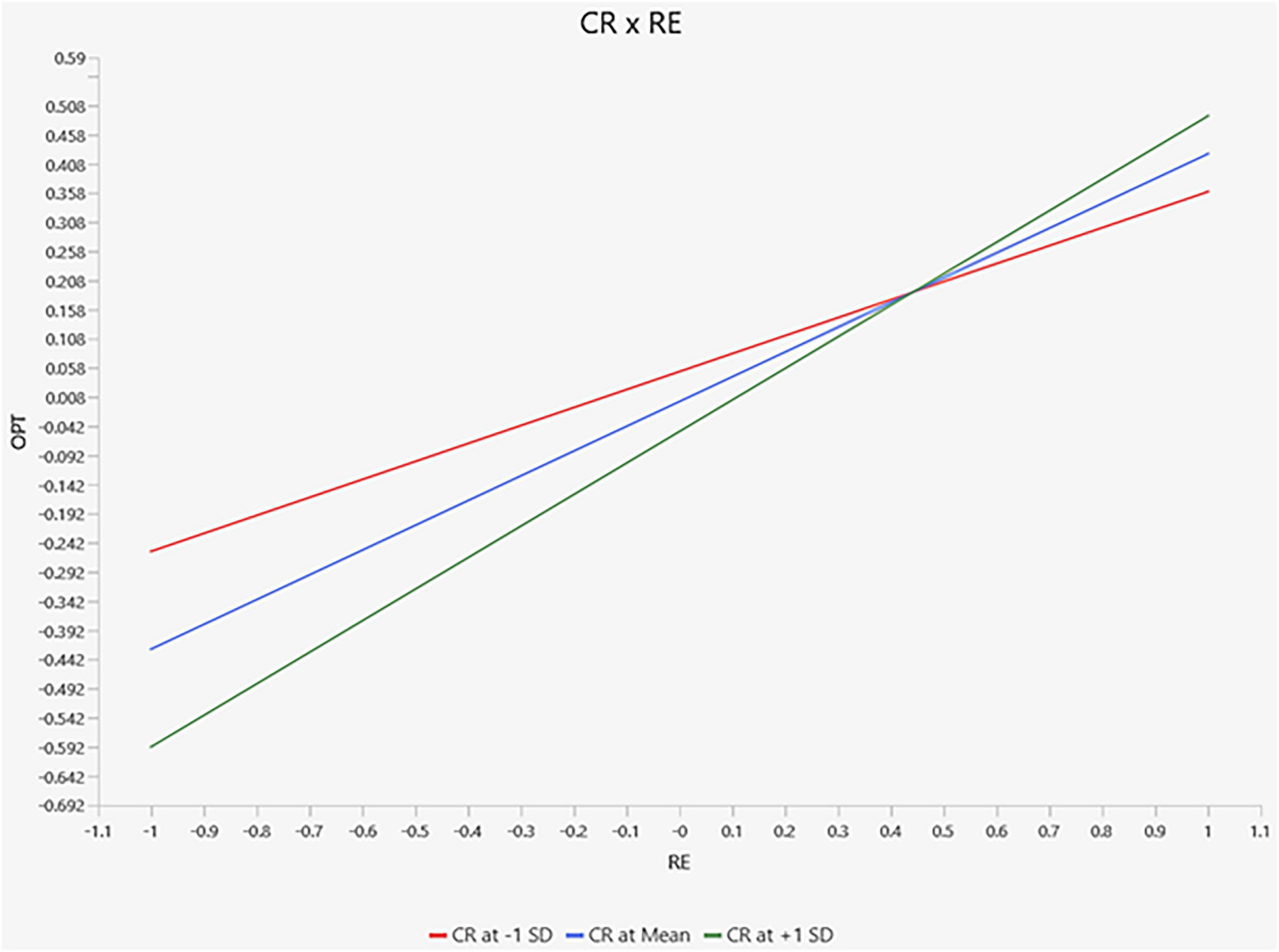
Moderation effect.

Figure 3 illustrates the simple slope analysis of the moderation effect. Although the difference between slopes for high and low cognitive reappraisal appears visually modest, the pattern indicates that the positive association between relational energy and optimism becomes stronger among students who frequently engage in cognitive reappraisal. This suggests that students who are more capable of reframing emotional experiences may derive greater cognitive benefits from energizing interpersonal interactions. The moderation effect should therefore be interpreted as a reinforcing mechanism rather than a large divergence in slopes.

**FIGURE 4:**
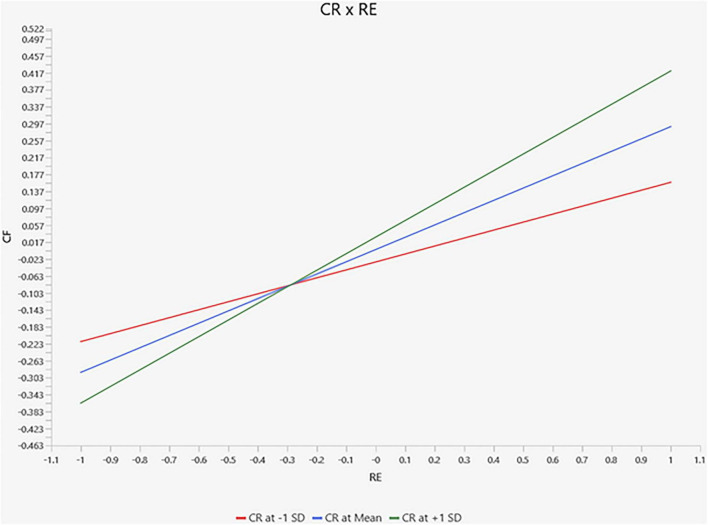
Moderated mediation effect.

Figure 4 presents the conditional indirect effect of relational energy on coping flexibility across different levels of cognitive reappraisal. The results indicate that the indirect pathway through optimism becomes stronger as cognitive reappraisal increases. However, the magnitude of this conditional effect remains relatively modest, suggesting that emotion regulation acts as a facilitating factor rather than a dominant determinant of the relational energy–coping relationship.

Although the hypothesized relationships are supported, alternative explanations cannot be ruled out. For example, individuals with higher levels of optimism may perceive interpersonal interactions as more energizing, or students with greater coping flexibility may be more likely to engage in positive social interactions. Future longitudinal or experimental research would be useful for disentangling these potential reciprocal relationships.

## 4 Discussion and conclusion

The primary objective of this study is to examine how relational energy contributes to students’ coping flexibility and to identify the psychological processes and boundary conditions that shape this relationship. The analysis demonstrates that this objective is achieved through the integration of optimism as a mediating mechanism and cognitive reappraisal as a key moderating factor. Relational energy shows a strong positive association with both optimism and coping flexibility, while optimism appears to represent a psychological pathway through which energized interpersonal interactions are associated with coping flexibility. The moderating role of cognitive reappraisal further reveals that students who habitually reinterpret emotional experiences gain amplified psychological advantages from relational energy, resulting in a stronger indirect effect on coping flexibility. Although the moderation and conditional indirect effects are modest in magnitude, such effects are common in psychological models that examine interpersonal and cognitive processes simultaneously (Aiken, 1991; Hayes, 2018). Even relatively small effects may accumulate over time in real-world academic contexts where multiple psychological resources interact to influence students’ coping behavior. Together, these findings enrich the understanding of how social, cognitive, and emotional processes interact to support adaptive coping in academic settings, offering valuable insights for fostering environments and skills that enhance students’ resilience and wellbeing.

### 4.1 Theoretical implications

The study presents several noteworthy theoretical implications by integrating relational energy, optimism, cognitive reappraisal, and coping flexibility into a unified explanatory framework. For instance, the first objective centers on establishing relational energy as a direct predictor of coping flexibility, and the support for H1 strengthens theoretical claims that energized interpersonal interactions enhance individuals’ adaptive coping capacities. Prior research indicates that relational energy promotes vitality, engagement, and psychological), and the present finding extends this stream of work by demonstrating its relevance beyond organizational settings. Similar observations in coping literature suggest that social resources broaden individuals’ behavioral repertoires in stressful situations (Fredrickson, 2004). By linking relational energy with coping flexibility in a student context, the study advances the understanding of how interpersonal uplift translates into cognitive–behavioral adaptability. Importantly, this relationship should also be viewed within broader interpersonal dynamics, as prior work suggests that social interactions can either energize or deplete individuals depending on relational quality and situational demands (Quinn et al., 2012). Thus, relational energy may represent a specific form of interpersonal resource that contributes to adaptive coping when interactions are experienced as supportive and psychologically uplifting.

The second objective examines whether relational energy enhances optimism, and the strong support for H2 aligns with the contention that positive relational experiences nurture favorable cognitive appraisals. Earlier work shows that relational energy increases emotional and psychological resourcefulness (Braha and Karabulut, 2023; Cabrera et al., 2025; Kennett et al., 2021), while optimism theory argues that supportive environments promote positive future expectations (Rossetti and Zlomke, 2021). The current finding therefore connects these previously separate strands of literature and demonstrates that the motivational strength derived from energized interactions meaningfully shapes students’ optimistic outlooks. This relationship is consistent with evidence showing that social affirmation and energizing interactions cultivate cognitive positivity (Easterbrook et al., 2021). However, the relationship between relational experiences and optimism may also operate in reciprocal ways. Individuals who possess more optimistic dispositions may interpret interpersonal encounters more positively and therefore perceive higher relational energy in their interactions (Carver and Scheier, 2014). Recognizing this possibility highlights the dynamic nature of interpersonal and cognitive processes and underscores the need for future longitudinal research.

The third objective evaluates optimism as a mediator, and the support for H3 confirms a theoretically coherent pathway in which relational energy enhances optimism, which in turn improves coping flexibility. Prior research indicates that optimism predicts adaptive coping behaviors and resilience (Gavín-Chocano et al., 2023; Smida et al., 2021). In addition, this notion is supported by broaden-and-build theory, which argues that positive cognitive states expand individuals’ coping options (Fredrickson, 2004). By situating relational energy as an interpersonal antecedent of optimism, the study enriches existing models by showing how energized social exchanges initiate upward cognitive spirals that culminate in flexible coping. At the same time, the magnitude of the mediation effect suggests that optimism represents one of several psychological mechanisms through which interpersonal vitality may influence coping behavior. Other mechanisms, such as emotional resilience or psychological capital, may also contribute to the translation of relational experiences into adaptive coping responses (Luthans et al., 2007).

The fourth objective investigates whether cognitive reappraisal strengthens the effect of relational energy on optimism, and the support for H4 demonstrates that adaptive emotion regulation significantly conditions this relationship. Previous studies consistently show that cognitive reappraisal enhances emotional wellbeing and promotes positive affective and cognitive outcomes (Abraham et al., 2023; Rodriguez et al., 2020). When combined with relational energy, the interaction suggests that individuals who habitually reinterpret stressful situations derive greater cognitive benefit from energizing social interactions. This aligns with evidence showing that emotion regulation amplifies the effect of social resources on psychological functioning (Brites et al., 2024), thereby extending the literature by identifying reappraisal as a boundary condition that magnifies the cognitive gains associated with relational energy. Nevertheless, the moderation effect observed in the study is relatively modest, suggesting that cognitive reappraisal acts as a reinforcing mechanism rather than a dominant determinant of optimism formation.

The final objective considers whether cognitive reappraisal strengthens the indirect effect of relational energy on coping flexibility through optimism. The support for H5 reveals a conditionally mediated process in which the benefits of relational energy are most pronounced for individuals who actively reshape their emotional interpretations. Research indicates that optimism and cognitive reappraisal jointly facilitate adaptive coping (Bai et al., 2025), yet their combined role within a relational energy framework has remained underexplored. This study advances theoretical understanding by showing that when students possess strong reappraisal tendencies, relational energy becomes a more powerful initiator of optimism and coping flexibility. However, the conditional indirect effect is comparatively small, indicating that the joint influence of relational energy and cognitive reappraisal should be interpreted as a subtle but theoretically meaningful mechanism contributing to coping flexibility. These insights integrate interpersonal, cognitive, and regulatory mechanisms into a more comprehensive framework that enriches positive psychology and coping theory.

### 4.2 Practical implications, limitations, and future research directions

The practical implications of this study are grounded in its demonstration that relational energy, optimism, and cognitive reappraisal operate jointly to enhance coping flexibility among students. These findings present several actionable insights for educators, counselors, and university administrators who aim to strengthen student wellbeing. For instance, because relational energy shows a strong positive influence on optimism and coping flexibility, institutions may prioritize interactional climates that promote energizing peer and teacher–student engagement. Programs that cultivate interpersonal connection, such as mentorship circles, collaborative learning spaces, and peer-support groups, may help students experience more relational vitality. Even modest improvements in interpersonal vitality may translate into meaningful psychological benefits when experienced repeatedly across students’ daily academic interactions.

More specifically, the moderated mediation findings suggest that interventions should not focus on relational support alone but should simultaneously develop students’ cognitive emotion regulation skills. Because cognitive reappraisal strengthens the effect of relational energy on optimism, universities may implement integrated programs that combine relational engagement with structured emotion-regulation training. For example, peer mentoring initiatives or collaborative learning environments could incorporate short reflective exercises that teach students how to reinterpret academic setbacks and interpersonal challenges in constructive ways. Such combined interventions may enable students to more effectively convert energizing interpersonal experiences into optimistic expectations and flexible coping responses.

In addition, optimism emerges as a key psychological mechanism linking relational experiences to adaptive coping. Rather than relying solely on general positive psychology interventions, universities may design targeted activities that explicitly connect social interaction with future-oriented thinking. For instance, group-based reflection sessions, resilience-building workshops, or guided peer discussions may help students translate supportive interactions into constructive interpretations of academic challenges. The moderating role of cognitive reappraisal further indicates that training in emotion regulation strategies, including cognitive reframing and structured reflection, can amplify the benefits of supportive social environments. Universities could integrate such skills into orientation programs, counseling services, or academic success courses to help students more effectively reinterpret stressors and convert relational experiences into adaptive coping. By aligning interpersonal engagement initiatives with emotion regulation training, institutions may more effectively activate the psychological mechanisms identified in the present study. Such initiatives may also support the development of personal mastery among students, which emphasizes continuous self-improvement, reflective learning, and sustained engagement with lifelong learning processes (McOwen et al., 2025). Overall, these implications highlight the value of combining interpersonal, cognitive, and emotional development initiatives to foster resilience and psychological flexibility in educational contexts.

Although the study offers valuable insights, several limitations warrant consideration. The reliance on self-report data may introduce common method variance, even though validated scales and procedural remedies help mitigate this concern. In addition, self-report surveys collected in face-to-face settings may be susceptible to social desirability bias, as participants may respond in ways that reflect socially favorable emotional or interpersonal behaviors. The cross-sectional design also limits causal inference, as relational energy, optimism, and coping flexibility unfold dynamically over time; therefore, the present findings should be interpreted as associative rather than causal relationships. Longitudinal or experimental research designs would be necessary to establish the temporal ordering of these variables and to confirm the causal mechanisms proposed in the model. It is also possible that individuals with stronger coping flexibility may engage more actively in positive interpersonal interactions, suggesting potential reciprocal dynamics among the variables examined.

Furthermore, the sample consists of university students, which may restrict generalizability to other populations such as working adults, adolescents, or clinical groups. Another important limitation concerns cultural specificity. Because the data were collected from Chinese university students, cultural norms related to interpersonal relationships, emotional expression, and coping strategies may shape how relational energy and cognitive reappraisal are experienced and interpreted. As such, the strength and nature of the observed relationships may differ in other cultural contexts. Cultural context presents another important limitation, as the experience and expression of relational energy or reappraisal may vary across collectivist and individualist settings. Future studies may address these constraints by employing multi-source data, incorporating time-lagged or diary methodologies, and testing cultural or demographic variations in the proposed relationships.

Drawing from these limitations, several directions emerge for future research. Scholars may explore additional mediating processes through which relational energy enhances coping, such as psychological capital, emotional resilience, or cognitive flexibility-related neural mechanisms. Examining alternative moderators, including social support quality, stress appraisal tendencies, or personality traits such as openness or emotional stability, may reveal deeper boundary conditions in the model. Future research may also benefit from employing longitudinal, multi-wave, or experience-sampling designs to capture how relational energy fluctuates across time and how these fluctuations influence students’ evolving coping responses. Longitudinal research could capture how relational energy fluctuates across academic semesters and how sustained interpersonal vitality shapes long-term development of coping skills. Cross-cultural comparisons may further clarify whether the magnitude of these effects differs across societies with varying norms regarding energy exchange, emotional expression, and coping expectations. Experimental intervention studies testing programs that simultaneously cultivate relational engagement and cognitive reappraisal skills would provide particularly valuable evidence for translating the theoretical mechanisms identified in this study into practical educational strategies. Finally, intervention-based studies testing whether relational energy training, optimism enhancement workshops, or reappraisal-focused programs effectively strengthen coping flexibility would provide meaningful applied extensions of the current model. Together, these future directions deepen the theoretical and practical relevance of relational energy and its psychological pathways, encouraging more integrative exploration across interpersonal, cognitive, and emotional domains.

## Data Availability

The raw data supporting the conclusions of this article will be made available by the authors, without undue reservation.

## Appendix

**APPENDIX TABLE 1:** Model fit comparison.

Model	Description	χ^2^	Df	χ^2^/df	CFI	TLI	RMSEA	SRMR
One-factor model	All items loaded on a single latent factor	3561.82	464	7.67	0.52	0.48	0.124	0.132
Two-factor model	RE + OPT combined; CR + CF combined	2318.64	463	5.00	0.71	0.67	0.095	0.108
Three-factor model	RE + OPT combined; CR separate; CF separate	1584.37	461	3.43	0.86	0.84	0.071	0.074
Four-factor model (hypothesized)	RE, OPT, CR, and CF modeled as distinct constructs	1096.52	458	2.39	0.94	0.93	0.052	0.046
